# Unilateral Lower Limb Edema as an Atypical Presentation of Thyrotoxicosis: A Case Report

**DOI:** 10.7759/cureus.112318

**Published:** 2026-07-09

**Authors:** Shagufta Parveen, Abdullah Khalid, Aaliya Abdul Gafoor, Rameesha Zafar, Tariq Karim

**Affiliations:** 1 General Practice, Jabal Sina Medical Centre, Ajman, ARE; 2 Internal Medicine, Jabal Sina Medical Centre, Ajman, ARE; 3 Medicine, Allama Iqbal Medical College, Lahore, PAK

**Keywords:** atrial fibrillation, management of thyrotoxicosis, peripheral edema, thyrotoxicosis, unilateral leg edema

## Abstract

Peripheral edema in thyrotoxicosis is uncommon and is typically associated with cardiac dysfunction. Unilateral edema without overt heart failure is rarely reported. We present the case of a 40-year-old female patient who presented with edema involving the right lower limb and suprapubic and genital regions. Investigations revealed thyrotoxicosis associated with atrial fibrillation (AF). The patient demonstrated significant clinical improvement following treatment with antithyroid therapy, beta-blocker, glucocorticoids, and supportive management. This case highlights the importance of considering thyroid dysfunction in the differential diagnosis of unexplained unilateral edema after exclusion of more common vascular, infectious, and systemic causes.

## Introduction

Thyrotoxicosis is a clinical syndrome of hypermetabolism resulting from excess circulating thyroid hormone levels, regardless of etiology. In contrast, hyperthyroidism is a clinical condition characterized by a sustained increase in the synthesis and secretion of thyroid hormones by the thyroid gland [1]. While the diagnosis of hyperthyroidism is often readily recognized based on classical clinical features of hypermetabolism, atypical presentations involving other organ systems have been well documented [2]. In contrast to the non-pitting edema seen in thyroid dermopathy [1,3,4], pitting edema is uncommon in hyperthyroidism. When present, it is often attributed to thyrotoxicosis-induced cardiac dysfunction [2,5,6]. Although peripheral edema in thyrotoxicosis is usually bilateral and often linked to cardiac dysfunction, rare reports have described limb edema in the absence of overt heart failure [7-10].

We report a patient with unilateral lower limb, suprapubic, and genital edema in the setting of biochemical thyrotoxicosis and atrial fibrillation (AF), highlighting an unusual presentation that may broaden the differential diagnosis of unilateral edema. Following treatment for thyrotoxicosis, the edema resolved with improvement in thyroid hormone levels.

## Case presentation

A 40-year-old woman presented to a primary care clinic with a three-day history of progressive swelling involving the lower abdomen and suprapubic region, extending to the right lower limb from the thigh down to the ankle. The patient reported associated numbness and tingling in the right lower limb. The swelling was not associated with pain, pruritus, rash, fever, nausea, vomiting, or genitourinary symptoms, such as dysuria or vaginal discharge. There was no recent history of insect bites, trauma, or similar previous episodes. The patient denied chest pain, shortness of breath, or recent prolonged immobilization or travel. Her last menstrual period was recent and regular. She was a known case of hypertension, previously on amlodipine 5 mg once daily, which she had discontinued one month before presentation. Her past surgical history was significant for an appendectomy. She reported no known drug allergies. There was no significant family history.

**Figure 1:**
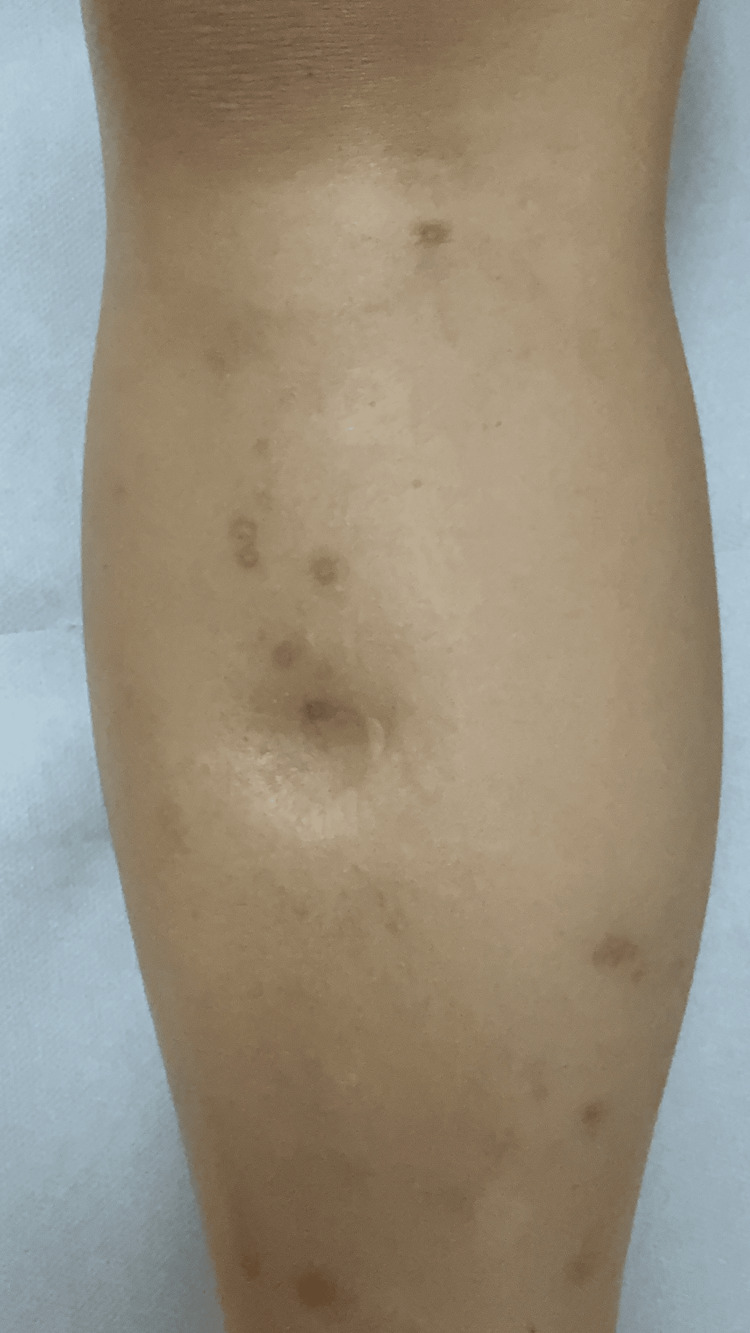
Unilateral pitting edema of the right lower limb at presentation

On examination, the patient was alert, oriented, and hemodynamically stable. Her pulse rate was 110 beats per minute, blood pressure was 150/90 mmHg, and temperature was 36.6 degrees Celsius. Examination revealed diffuse swelling involving the lower abdomen, including the suprapubic region and external genitalia, extending to the right lower limb from the thigh to the ankle. The overlying skin in the suprapubic and genital regions appeared tense and stretched without erythema. On palpation, the overlying skin was smooth, dry, with temperature equal to the surrounding skin, and non-tender. The edema in this region was non-pitting. In contrast, examination of the right lower limb demonstrated pitting edema extending from the thigh to the ankle, without associated overlying erythema or localized tenderness. No skin discoloration or rash was noted (Figure 1).

Neurological examination revealed normal power, tone, and reflexes with no focal neurological deficits. Bilateral inguinal lymph node examination revealed no palpable lymphadenopathy. Examination of the left lower limb was unremarkable, with no evidence of edema or skin changes. Abdominal examination revealed a soft, non-tender abdomen with no organomegaly or palpable masses. Cardiovascular examination revealed normal heart sounds with no audible murmurs and no evidence of raised jugular venous pressure. Respiratory examination demonstrated equal air entry bilaterally with no abnormal breath sounds. A gynecological evaluation was performed to assess for local causes of genital swelling; findings were unremarkable with no identifiable pathology.

Initial investigations were undertaken to evaluate the cause of the unilateral edema and the associated symptoms. These included a complete blood count (CBC), liver function tests (LFTs), renal function tests (RFTs), thyroid-stimulating hormone (TSH), and urinalysis. Given the diagnostic uncertainty at presentation, empirical therapy with corticosteroids, an antihistamine, and antibiotics was initiated for a presumed inflammatory or infectious etiology.

The following day, a review of laboratory results revealed a suppressed TSH level. Other baseline investigations, including CBC, RFT, and urinalysis, were within normal limits, except for an isolated elevation in alkaline phosphatase (ALP) (Table 1).

**Table 1:** Baseline laboratory investigations at presentation TSH: thyroid-stimulating hormone, FT3: free triiodothyronine, FT4: free thyroxine, TPO: thyroid peroxidase, WBC: white blood cell count, MCV: mean corpuscular volume, ALP: alkaline phosphatase, AST: aspartate aminotransferase, ALT: alanine aminotransferase, HPF: high-power field

Parameter	Result	Unit	Reference range
TSH	<0.005	µIU/mL	0.27-4.20
FT3	6.3	pg/mL	2.6-4.4
FT4	3.0	ng/dL	0.93-1.7
TPO antibodies	516.0	IU/mL	<34.0
WBC	6.63	×10³/µL	4.0-10.0
Hemoglobin	12.2	g/dL	12.0-15.0
MCV	75.8	fL	83.0-101.0
Platelets	137.0	×10³/µL	150.0-410.0
Random blood sugar	118.78	mg/dL	<140.0
Total bilirubin	0.69	mg/dL	0.1-1.2
ALP	304.88	U/L	30.0-120.0
Albumin	4.03	g/dL	3.5-5.3
AST	26.7	U/L	≤31.0
ALT	28.7	U/L	≤34.0
Urea	23.82	mg/dL	17.0-43.0
Creatinine	0.47	mg/dL	0.50-0.90
Urine protein	Absent	-	Absent
Urine WBC	2-3/HPF	-	0-5/HPF
Urine nitrite	Negative	-	Negative
Troponin I	0.009	ng/mL	Negative: <0.04, positive: ≥0.5

At the same visit, the patient reported new-onset palpitations and tremors, prompting further evaluation with electrocardiography (ECG) and serum troponin. The ECG revealed atrial fibrillation, while troponin levels remained within normal limits (Table 1).

Thyroid function tests revealed a suppressed TSH level of <0.005 uIU/mL (reference range: 0.27-4.20 uIU/mL), with an elevated free triiodothyronine (FT3) level at 6.3 pg/mL (reference range: 2.6-4.4 pg/mL) and free thyroxine (FT4) level at 3.0 ng/dL (reference range: 0.93-1.7 ng/dL). Thyroid peroxidase antibody levels were elevated at 516 IU/mL (reference range: <34 IU/mL), raising the possibility of an underlying autoimmune thyroid disorder. Following these findings, a targeted neck examination was performed that revealed no palpable thyroid enlargement, nodules, or bruit. There were no clinical features of thyroid eye disease.

Following the identification of biochemical thyrotoxicosis and atrial fibrillation, the patient was referred for specialist evaluation. Given the presence of unilateral limb edema, further evaluation for thromboembolic causes was undertaken. A focused point-of-care ultrasonographic assessment of the cardiac structures and proximal deep veins was performed, which did not demonstrate features suggestive of deep vein thrombosis, ventricular dysfunction, or pericardial effusion. Serum D-dimer and electrolyte levels were within normal limits (Table 2).

**Table 2:** D-dimer and electrolyte levels during further evaluation FEU: fibrinogen equivalent unit

Parameter	Result	Unit	Reference range
D-dimer	0.35	mg/L FEU	0.00-0.50
Sodium	137.3	mmol/L	135.0-146.0
Potassium	3.80	mmol/L	3.50-5.20
Chloride	97.7	mmol/L	97.0-109.0
Ionized calcium	1.13	mmol/L	1.10-1.29

In view of biochemical evidence of thyrotoxicosis in association with atrial fibrillation, the patient was commenced on carbimazole 15 mg three times daily, along with systemic corticosteroids (prednisolone) to mitigate peripheral thyroid hormone effects, and bisoprolol 2.5 mg once daily for rate control. Furosemide was administered for symptomatic relief of edema.

At one-week follow-up, the patient reported symptomatic improvement with a reduction in edema, tremors, and palpitations. Despite this early clinical improvement, repeat TFT levels remained abnormal, with persistently suppressed TSH and elevated FT3 and FT4 levels (Table 3). A repeat ECG demonstrated persistent atrial fibrillation. Following early clinical improvement in edema, prednisolone and furosemide were discontinued after a five-day course, while carbimazole and bisoprolol were continued.

**Table 3:** Thyroid function tests at one-week follow-up demonstrating persistent biochemical thyrotoxicosis despite early clinical improvement TSH: thyroid-stimulating hormone, FT3: free triiodothyronine, FT4: free thyroxine

Parameter	Result	Unit	Reference range
TSH	0.011	µIU/mL	0.27-4.20
FT3	12.66	pg/mL	1.8-4.2
FT4	6.36	ng/dL	0.5-1.4

**Figure 2:**
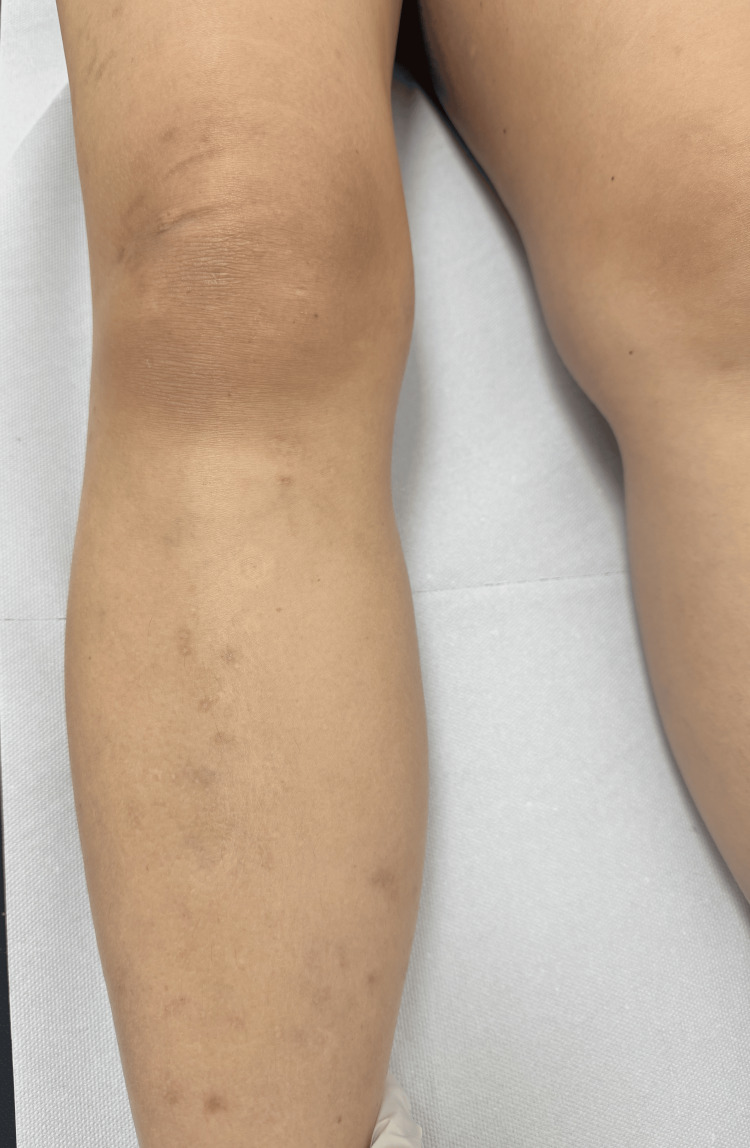
Near-complete resolution of right lower limb edema at four-week follow-up

At approximately four weeks of follow-up, the patient demonstrated near-complete resolution of edema, as shown in Figure 2, with no residual tremors or palpitations. Repeat TFTs performed during follow-up demonstrated biochemical improvement, with a reduction in FT3 and FT4 levels toward the reference range, as shown in Table 4. However, TSH levels remained suppressed, consistent with the expected delayed recovery of the hypothalamic-pituitary-thyroid axis. The patient subsequently continued follow-up with an endocrinologist, who advised continuation of carbimazole and bisoprolol; follow-up ECG data were not available. 

**Table 4:** Repeat thyroid function tests at four-week follow-up TSH: thyroid-stimulating hormone, FT3: free triiodothyronine, FT4: free thyroxine

Parameter	Result	Unit	Reference range
TSH	0.001	µIU/mL	0.38-5.33
FT3	3.63	pg/mL	2.47-3.90
FT4	1.15	ng/dL	0.61-1.11

## Discussion

Thyrotoxicosis refers to the clinical state resulting from excess circulating thyroid hormone levels, regardless of etiology, whereas hyperthyroidism specifically denotes increased synthesis and secretion of thyroid hormone by the thyroid gland [11,12]. In this case, suppressed TSH with elevated FT3 and FT4 confirmed thyrotoxicosis, while markedly elevated thyroid peroxidase antibodies suggested an underlying autoimmune etiology. However, the absence of classical clinical features such as goiter, ophthalmopathy, or thyroid bruit highlights the variable and sometimes atypical clinical spectrum of thyrotoxicosis [11,13]. Further diagnostic evaluation, including TSH receptor antibody testing, radionuclide uptake studies, and thyroid ultrasonography, was not performed. Therefore, definitive etiologic classification was not possible, and both Graves’ disease and alternative causes, including silent thyroiditis or other forms of autoimmune thyroiditis, remain possible.

The most striking feature of this presentation was unilateral edema involving the right lower limb and genital region. Peripheral edema in thyrotoxicosis is uncommon and is typically bilateral, most often associated with high-output cardiac failure or thyrotoxic cardiomyopathy [2,5,6]. Similar cases of edema in hyperthyroidism without cardiac failure have been described, although unilateral involvement has been only rarely reported in the literature [7-10]. The mechanisms underlying edema formation in thyrotoxicosis without heart failure are not fully defined and are likely multifactorial. Thyroid hormone excess leads to peripheral vasodilation with reduced systemic vascular resistance, resulting in neurohormonal activation and sodium-water retention [5,12,13]. Thyroid hormone excess has also been proposed to activate the renin-angiotensin-aldosterone system by increasing renal renin synthesis, potentially contributing to fluid retention and edema [9]. Increased capillary permeability and altered lymphatic drainage may further contribute to interstitial fluid accumulation [14]. Although the exact pathophysiology of unilateral edema remains unclear, localized vascular or lymphatic mechanisms have been proposed in previously reported cases [7-10]. In the present patient, the coexistence of non-pitting edema in the suprapubic and genital regions with pitting edema of the lower limb may reflect overlapping vascular and interstitial mechanisms.

The unilateral distribution initially raised concern for deep venous thrombosis, cellulitis, lymphatic obstruction, or pelvic pathology. Although there were no clinical features suggestive of deep venous thrombosis or infection, the D-dimer was normal, and bedside ultrasonographic assessment was unremarkable, these conditions could not be definitively excluded. In particular, as formal venous Doppler ultrasonography and dedicated pelvic or abdominal venous imaging were not performed, a small proximal thrombus or another cause of venous or lymphatic obstruction cannot be completely excluded. These limitations were mainly due to resource constraints in a primary care setting. Clinical evaluation also did not suggest overt cardiac, renal, or hepatic dysfunction. The marked clinical improvement in edema following the initiation of treatment may suggest a possible association between thyrotoxicosis and the patient’s clinical presentation.

Atrial fibrillation (AF) was an important associated finding in this case. AF is one of the most common cardiovascular manifestations of thyrotoxicosis, occurring in approximately 10%-25% of patients [5,15]. Excess thyroid hormone increases beta-adrenergic receptor sensitivity, shortens atrial refractory periods, and promotes atrial electrical remodeling, predisposing to arrhythmogenesis [5,15]. AF with rapid ventricular response may also have contributed to the patient’s altered hemodynamic state [5], although overt heart failure was not clinically evident.

Beta-blockers remain a key component of symptomatic management in thyrotoxicosis. While propranolol is commonly used for its additional effect on the peripheral conversion of FT4 to FT3 [13,16,17], cardio-selective agents such as atenolol and bisoprolol may be preferred in patients with atrial fibrillation because of their longer duration of action and effective rate control [15,17]. In this case, bisoprolol provided effective control of palpitations and tremors. It is also important to note that the patient initially received glucocorticoids before confirmation of biochemical hyperthyroidism. In addition to their anti-inflammatory effects, glucocorticoids are known to reduce peripheral conversion of FT4 to FT3 [13,16] and may therefore have contributed to the early improvement in edema.

An isolated elevation in alkaline phosphatase (ALP) was also observed. In thyrotoxicosis, such elevation is most commonly attributed to increased bone turnover rather than hepatobiliary disease, particularly when transaminases and bilirubin remain normal [12,13]. Thyroid hormones enhance both osteoclastic and osteoblastic activity, leading to accelerated skeletal remodeling and increased release of bone-derived ALP [18].

Another important observation in this case was the delayed normalization of TSH and persistence of atrial fibrillation despite early symptomatic and biochemical improvement. Suppression of TSH may persist for several weeks to months after normalization of thyroid hormone levels due to delayed recovery of the hypothalamic-pituitary-thyroid axis [11,12]. Similarly, atrial fibrillation may take several weeks to revert to sinus rhythm even after biochemical euthyroidism is achieved [15,19].

## Conclusions

Overall, this case describes an unusual presentation of unilateral lower limb, lower abdominal, suprapubic, and genital edema occurring in association with biochemical thyrotoxicosis and atrial fibrillation, without clinical evidence of heart failure. The marked clinical improvement following treatment may suggest a possible association between thyrotoxicosis and the patient’s clinical presentation. This case emphasizes that thyroid dysfunction should be considered in the differential diagnosis of unexplained unilateral edema after appropriate evaluation of more common vascular, infectious, and systemic causes. As a single case, these findings cannot be generalized; however, further reports may help determine whether this represents an underrecognized clinical presentation associated with thyrotoxicosis or a rare association.

## References

[1] Braverman LE, Cooper DS (2013). Introduction to thyrotoxicosis. Werner & Ingbar's The Thyroid: A Fundamental and Clinical Text.

[2] Boxall EA, Lauener RW, McIntosh HW (1964). Atypical manifestations of hyperthyroidism. Can Med Assoc J.

[3] Dhali TK, Chahar M (2014). Thyroid dermopathy-a diagnostic clue of hidden hyperthyroidism. Dermatoendocrinol.

[4] Cohen B, Cadesky A, Jaggi S (2023). Dermatologic manifestations of thyroid disease: a literature review. Front Endocrinol (Lausanne).

[5] Osuna PM, Udovcic M, Sharma MD (2017). Hyperthyroidism and the heart. Methodist Debakey Cardiovasc J.

[6] Yakasai BA, Yakasai HB (2018). A case report on congestive cardiac failure due to hyperthyroidism. J Clin Trial Cardiol.

[7] Volke V, Matjus S (2012). Unilateral pitting edema of the leg as a manifestation of Graves' disease: a case report. J Med Case Rep.

[8] Kang J, Frederick Nelson N (2024). A rare presentation of hyperthyroidism: T3 thyrotoxicosis related to bilateral pitting edema of the leg. J Clin Images Med Case Rep.

[9] Kazama I, Mori Y, Baba A, Nakajima T (2014). Pitting type of pretibial edema in a patient with silent thyroiditis successfully treated by angiotensin ii receptor blockade. Am J Case Rep.

[10] Bostan H, Uçan B, Kızılgül M, Düğer H, Akhanlı P, Çakal E (2021). A rare presentation of Graves’ disease: bilateral pitting edema of the legs. J Med Palliat Care.

[11] Korem Kohanim Y, Milo T, Raz M (2022). Dynamics of thyroid diseases and thyroid-axis gland masses. Mol Syst Biol.

[12] Ross DS, Burch HB, Cooper DS (2016). 2016 American Thyroid Association guidelines for diagnosis and management of hyperthyroidism and other causes of thyrotoxicosis. Thyroid.

[13] Idrose AM (2015). Acute and emergency care for thyrotoxicosis and thyroid storm. Acute Med Surg.

[14] Goyal A, Singh B, Afzal M (2025). Peripheral edema. StatPearls [Internet].

[15] N J, Francis J (2005). Atrial fibrillation and hyperthyroidism. Indian Pacing Electrophysiol J.

[16] De Almeida R, McCalmon S, Cabandugama PK (2022). Clinical review and update on the management of thyroid storm. Mo Med.

[17] Szybiak-Skora W, Miedziaszczyk M, Szałek E, Lacka K (2025). The therapeutic potential of propranolol and other beta-blockers in hyperthyroidism. Int J Mol Sci.

[18] Reddy PA, Harinarayan CV, Sachan A, Suresh V, Rajagopal G (2012). Bone disease in thyrotoxicosis. Indian J Med Res.

[19] Reddy V, Taha W, Kundumadam S, Khan M (2017). Atrial fibrillation and hyperthyroidism: a literature review. Indian Heart J.

